# Advances in Gold Nanoparticles for the Diagnosis and Management of Alzheimer’s Disease

**DOI:** 10.3390/pharmaceutics17091158

**Published:** 2025-09-03

**Authors:** Bhagavathi Sundaram Sivamaruthi, Periyanaina Kesika, Natarajan Sisubalan, Chaiyavat Chaiyasut

**Affiliations:** 1 Office of Research Administration, Chiang Mai University, Chiang Mai 50200, Thailand; sivamaruthi.b@cmu.ac.th (B.S.S.); kesika.p@cmu.ac.th (P.K.); 2 Innovation Center for Holistic Health, Nutraceuticals, and Cosmeceuticals, Faculty of Pharmacy, Chiang Mai University, Chiang Mai 50200, Thailand; 3 Department of Microbiology, Karpagam Academy of Higher Education, Coimbatore 641021, India; 4 PG and Research Department of Botany, Pachaiyappa’s College, Affiliated to University of Madras, Chennai 600030, India

**Keywords:** Alzheimer’s disease, functionalized gold nanoparticles, gut–brain axis, amyloid β fibrillation, blood–brain barrier

## Abstract

Alzheimer’s disease (AD) presents a significant challenge in modern healthcare, prompting exploration into novel therapeutic strategies. This review clearly classifies different types of gold (Au) nanoparticles (NPs) (AuNPs), links them to the gut–brain axis, highlights recent advances, and points out future research needs, offering a more updated perspective than earlier reviews. Diverse approaches have emerged from single to hybrid and functionalized AuNPs, including innovative nanotherapeutic agents like Au nanorods-polyethylene glycol-angiopep-2 peptide/D1 peptide and noninvasive dynamic magnetic field-stimulated NPs. AuNPs have been reported for the neuroprotective properties. Clinical applications of AuNPs highlight their promise in diagnosis and therapeutic monitoring. However, challenges persist, notably in overcoming blood–brain barrier limitations and refining drug delivery systems. Furthermore, the incomplete understanding of AD’s physiological and pathological mechanisms hinders therapeutic development. Future research directions should prioritize elucidating these mechanisms and optimizing AuNPs physicochemical properties for therapeutic efficacy. Despite limitations, nanomaterial-based therapies hold promise for revolutionizing AD treatment and addressing other central nervous system disorders. It also emphasizes the importance of further investigation into the potential of AuNPs, envisioning a future where they serve as a cornerstone in advancing neurological healthcare.

## 1. Introduction

Alzheimer’s disease (AD) is a weakening condition that primarily affects older individuals, marked by gradual and progressive deterioration of the brain, attributed to the accumulation of plaques in the hippocampus [1]. A series of studies suggests that the formation of plaques initiates approximately two decades before the appearance of clinical symptoms, leading to uncertainty regarding the precise progression of pathologies associated with AD [2,3]. AD prevalence is on the rise globally [4]. Currently, an estimated 6.9 million Americans aged 65 years and older are affected by AD, and this number is projected to increase to approximately 13.8 million by 2060 in the absence of effective preventive or curative interventions [5]. Medications designed to alleviate cognitive decline in AD work by either targeting neurotransmitters or modulating enzymes, and they are administered intranasally to facilitate direct delivery to the brain [6]. Nevertheless, the utilization of these medications has led to frequent instances of therapy failure attributed to challenges such as insufficient absorption, neuronal cell membranes, and brain toxicity, besides various pharmacodynamic and pharmacokinetic factors [7,8].

**Figure 1:**
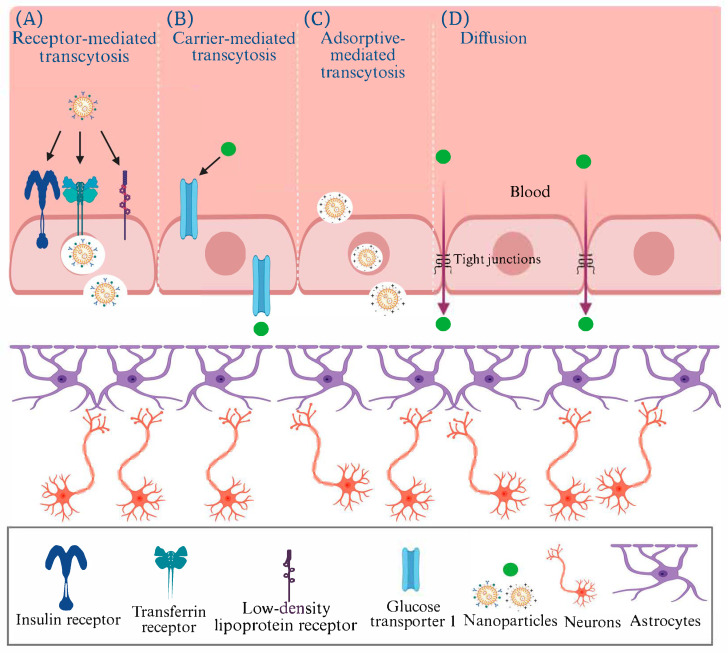
The methods of transport by nanomedicine through the blood–brain barrier via (**A**) receptor-mediated transcytosis, (**B**) carrier-mediated transcytosis, (**C**) adsorptive-mediated transcytosis, (**D**) Diffusion (Recreated with permission from Navarro Martínez et al., 2023 [29] under the https://creativecommons.org/licenses/by/4.0/deed.en (accessed on 31 July 2025); Created in BioRender.com).

Nanoparticles (NPs) and nanocomposites can be used to treat injuries and facilitate drug delivery [9,10]. Through clinical and preclinical studies, nanomedicines/NPs are recognized for their potential to treat and manage several diseases and disorders [11,12,13,14]. Utilizing engineered NPs with distinct physicochemical characteristics and the ability to cross the blood–brain barrier (BBB) holds promise as a potential strategy for addressing pharmacological and biomedical challenges in treating brain disorders, such as AD [15]. The primary benefit of nanomedicines in treating brain disorders such as AD lies in their ability to release drugs in a controlled manner at specific sites [16,17]. Nanomedicine approaches for AD are interested in employing metallic nanoparticles for targeted drug delivery across the BBB. The synthesis of metallic nanoparticles using chemistry-based methods has some limitations, even though certain metallic nanoparticles, including cerium, selenium, gold, and iron, are recognized for their notable anti-AD properties [18]. Nanomedicine-based theranostic formulations for AD were studied, which include AuNPs [19,20,21], protein-coated NPs [22,23,24], and antibody (Ab)-decorated NPs [25,26,27,28]. Figure 1 illustrates the principal mechanisms by which NPs can traverse the BBB to reach the central nervous system. The BBB, formed by endothelial cells joined by tight junctions and supported by astrocytes, restricts paracellular transport while permitting selective entry through specialized pathways. In receptor-mediated transcytosis, NPs functionalized with ligands such as transferrin, insulin, or apolipoproteins bind to endothelial receptors (e.g., transferrin, insulin, or LDL receptors), undergo endocytosis, and are transported across the cell into the brain. Carrier-mediated transcytosis exploits endogenous transporters, such as glucose transporter 1, to deliver molecules that mimic natural substrates like glucose or amino acids. Adsorptive-mediated transcytosis occurs through electrostatic interactions between cationic NPs and negatively charged endothelial surfaces, leading to nonspecific uptake and transcytosis. In contrast, passive diffusion is limited to small, lipophilic molecules that can cross endothelial membranes directly, as tight junctions restrict paracellular movement. Collectively, these pathways highlight how engineered nanocarriers can be designed to exploit specific transport routes for targeted drug delivery across the BBB.

AuNPs are most notably significant in facilitating drug delivery from the bloodstream beyond the BBB to the brain, particularly for addressing neurodegenerative illnesses. Several formulations of AuNPs are employed in diagnostic and therapeutic approaches for treating AD [30]. In a mouse model of AD, AuNPs stabilized with d-glutathione can effectively penetrate the BBB after intravenous administration [31]. These nanoparticles exhibit potent repressing effects against amyloid-beta (Aβ)_42_ aggregation without inducing neurotoxicity [31]. The administration of AuNPs through injections directly into the hippocampus and the peritoneum may enhance spatial learning and memory [32]. A method has been developed to enhance the defensive effects of dietary polyphenolic composites, such as anthocyanins, by linking them to AuNPs. Research indicates that administering anthocyanin-loaded polyethylene glycol-AuNPs to mice with an AD model shows potential to delay age-related neurodegenerative conditions [33]. Administering AuNPs anchored with maize tetrapeptide can enhance the functioning of the central cholinergic system while decreasing acetylcholinesterase levels, which implies that the newly identified tetrapeptide prevents AD as a neuroprotective agent [34]. Likewise, administering AuNPs to animals with AD has effectively alleviated AD symptoms by decreasing neuroinflammation and regulating mitochondrial functions [35].

**Figure 2:**
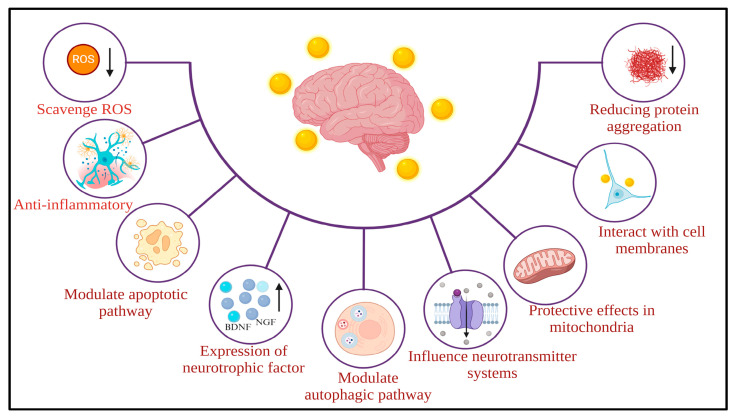
The illustration depicts the possible molecular and cellular mechanisms associated with the neuroprotective effects of AuNPs. ↑ = Increase; ↓ = Decrease (Adapted from Chiang et al., 2024 [36] under the https://creativecommons.org/licenses/by/4.0/deed.en (accessed on 31 July 2025).

The neuroprotective role of AuNPs involves complex interactions at both the molecular and cellular levels, encompassing various molecular mechanisms [36] (Figure 2).

As investigations continue, numerous potential mechanisms have been proposed for the neuroprotective effects of AuNPs, drawing on preclinical research [36]. One proposed mechanism is that AuNPs may have intrinsic antioxidant properties, potentially contributing to their neuroprotective effects [37]. AuNPs can counteract reactive oxygen species (ROS) and reduce oxidative stress, a crucial factor in the development of neurodegenerative conditions [38]. Additionally, AuNPs may have anti-inflammatory properties by influencing various signaling pathways [39]. AuNPs can potentially inhibit the activation of microglia and astrocytes, thereby reducing the release of pro-inflammatory cytokines such as interleukin-1 beta (IL-1β) and tumor necrosis factor-alpha (TNFα). The improvement of neuroinflammation plays a critical role in neuroprotection. Furthermore, AuNPs have demonstrated the capacity to influence apoptotic pathways, thereby contributing to neuroprotection [37]. They may affect key regulators of apoptosis, including the Bcl-2 family, caspases, and other proteins, thereby enhancing cell survival and inhibiting neuronal apoptosis in neurodegenerative disorders [40].

Mitochondrial dysfunction is a common feature in numerous neurological disorders. AuNPs may offer shielding effects on mitochondria, preserving their function and blocking the release of harmful reactive species, thus contributing to the maintenance of cellular balance [41]. Additionally, AuNPs could interact with cell membranes, affecting their fluidity and stability. It might impact intracellular signaling pathways, leading to protective responses. Moreover, AuNPs may impede the aggregation of misfolded proteins, such as Aβ in AD or α-synuclein in Parkinson’s disease [41].

While earlier reviews have discussed the role of AuNPs in neurodegenerative diseases, the present review provides several new perspectives. We provide a clear classification of AuNPs into single, dual-metal, hybrid, and functionalized forms, explaining their different mechanisms and applications. In addition, the present review includes the most recent advances, such as functionalized AuNPs, photothermal and magnetic field-based therapies, and biosensors for early diagnosis.

## 2. Pathology of Alzheimer’s Disease

Although significant advancements have been made in understanding AD, its underlying mechanisms of pathogenesis remain complex. A series of studies has highlighted the critical role of oxidative stress in AD pathology, triggering processes such as inflammation, protein aggregation, mitochondrial damage, and overall cellular dysfunction, leading to neuronal injury [42,43,44,45]. Furthermore, in 2018, researchers proposed a framework for advancing the concept of AD as a biological disease [46]. The deposition and aggregation of Aβ peptides into extracellular plaques and the establishment of intraneuronal knots containing hyperphosphorylated tau protein are the main pathological features of AD. Nevertheless, the precise connections between glial cell responses, amyloidogenic protein aggregation, neuronal death, synaptic dysfunction, and cognitive decline remain unclear [47].

Mutations in the genes responsible for amyloid precursor protein (APP), presenilin 1 and 2 (PSEN1, PSEN2) are implicated in the development of early-onset AD [48,49], while factors like aging, diet, lifestyle, and apolipoprotein E4 gene expression exacerbate late-onset AD [50]. The Aβ peptide, derived from APP by proteolytic cleavage [51], undergoes intricate processing within the cell, influenced by its localization and enzymatic activities. Initially transported from the trans-Golgi network, APP can be directed to the cell surface or the endosomal compartment. At the cell surface, it follows a non-amyloidogenic pathway characterized by α-secretase cleavage within the Aβ domain, yielding soluble APPα (sAPPα) and a membrane-bound C-terminal fragment. Subsequent cleavage by γ-secretase generates a P3 fragment and the APP intracellular domain (AICD). Conversely, within the endosomal compartment, APP enters the amyloidogenic pathway. Here, β-secretase acts on the extracellular domain, releasing sAPPβ and a membrane-bound C-terminal fragment of 99 amino acids. Further cleavage by γ-secretase releases AICD and soluble Aβ fragments [52].

In AD pathogenesis, the Aβ peptide undergoes oligomerization in the presence of transition metal ions like Fe^2+^ and Cu^2+^, leading to lipid peroxidation and subsequent generation of 4-hydroxynonenal [53]. Glucose and glutamate transport impairment exacerbates calcium influx, triggering the production of inositol 1,4,5-trisphosphate and subsequent calcium release from endoplasmic reticulum stores [54]. This calcium influx activates calpains, activating cyclin-dependent kinase 5, resulting in tau hyperphosphorylation. This event leads to the formation of neurofibrillary tangles, which disrupt microtubules and impair axonal transport, ultimately causing neuronal and synaptic dysfunction and subsequent neuronal death [55].

**Figure 3:**
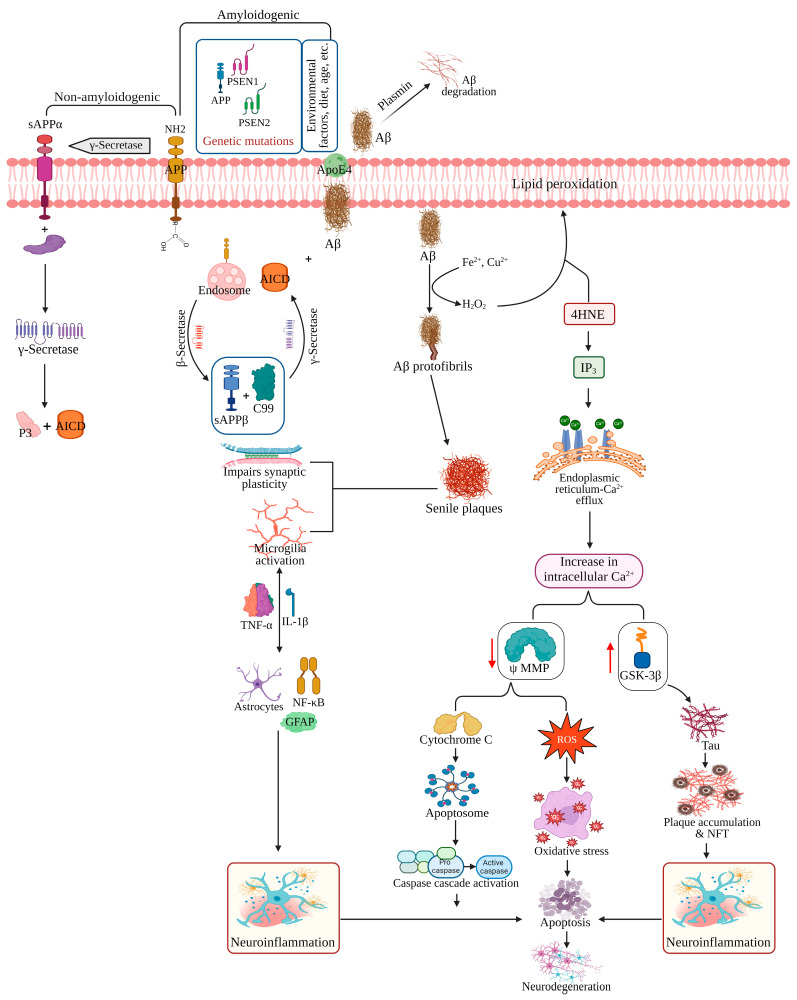
A diagram illustrating the various factors contributing to the risk of Alzheimer’s disease (AD), the metabolism of Amyloid precursor protein (APP), and the pathways leading to the development of AD. 4HNE: 4-hydroxynonenal; AICD: APP intracellular domain; ApoE4: Apolipoprotein E4; C99: Membrane-bound 99 amino acid comprising CTF-β; GFAP: Glial fibrillary acidic protein; GSK-3β: Glycogen synthase kinase-3 Beta; IL-1β: Interleukin-1 beta; IP_3_: inositol 1,4,5-trisphosphate; NF-κB: Nuclear factor kappa-light-chain-enhancer of activated B cells; NFT: Neurofibrillary Tangles; PSEN1: Presenilin 1; PSEN2: Presenilin 2; ROS: Reactive oxygen species; sAPPα: Soluble APPα; sAPPβ: Soluble APPβ; TNFα: Tumor necrosis factor-alpha; ΨMMP: Mitochondrial membrane potential. (Recreated with permission from Kesika et al., 2021 [59] and Elsevier (License number: 5776460676484 dated 26 April 2024; Created in BioRender.com)).

Increased Ca^2+^ influx into mitochondria, along with ROS, triggers the formation of the high-conductance mitochondrial permeability transition pore associated with cyclophilin D, leading to the release of proapoptotic factors such as cytochrome c and apoptosis-inducing factor, subsequently activating the caspase cascade and resulting in neuronal apoptosis [56,57]. Additionally, Aβ plaques stimulate microglial cells to release IL-1, IL-6, IL-8, TNFα, and macrophage inflammatory protein-1, which in turn activate astrocytes to release cytokines, chemokines, and acute-phase proteins, further activating microglial cells [58]. Additionally, Aβ plaques activate microglia and astrocytes, releasing cytokines and chemokines and amplifying the inflammatory response in AD [59]. Multiple lines of evidence have revealed that AD is caused by the combined effect of several hypotheses, as illustrated in Figure 3.

## 3. Gold Nanoparticles, Gut–Brain Axis, and Microbiome

The concept of the gut–brain axis (GBA) has emerged to depict the communication network between the gut, its microbiota, and the brain. The GBA system encompasses a complex interplay of neural, endocrine, immune, and metabolic pathways crucial for maintaining brain homeostasis [60]. Molecular investigations have revealed a sophisticated interaction system that facilitates optimal gastrointestinal balance [61]. GBA regulates and synchronizes gut functions and the brain’s cognitive and functional domains. Neurological, immune, and endocrine messengers are pivotal in orchestrating communication within the GBA. Components of GBA include the central nervous system (CNS), autonomic nervous system, hypothalamic–pituitary–adrenal axis, and enteric nervous system, forming an intricate network [62].

It has been reported that the gut microbiome may play a crucial role in the onset and progression of AD [59]. The gut microflora produces substances such as neuromodulators or neurotransmitters (like choline, tryptophan, and short-chain fatty acids (SCFAs)) as well as hormones (such as ghrelin and leptin), which were influenced by various internal factors like metabolites, genetics, hormones, and immune responses, also external factors such as lifestyle, infections, and diet. Subsequently, these neurotransmitters and neuromodulators regulate the CNS [63].

Exploring the potential of AuNPs to influence the GBA and modulate the microbiome presents an innovative frontier in biomedical research. Advanced tools and methodologies are crucial for enhancing brain visualization and evaluating drug permeability across the BBB [64]. An emerging and promising area in nanomedicine explores the use of AuNPs to advance novel approaches in the preclinical management of brain-related conditions, such as neurodegenerative diseases and cerebral tumors. The efficacy of AuNPs in treating various brain disorders is hampered by the selective permeability of the BBB, which firmly regulates the entry of substances into the brain. Peptides and proteins emerge as potential tools to enhance the delivery of AuNPs to the CNS. Peptide-capped nanoparticles can cross the BBB through various mechanisms, including receptor-mediated transcytosis, active transport, cell-mediated pathways, or a synergistic combination [65].

The studies on the impacts of AuNPs on gut microbial profiles are limited. In contrast, recent studies have explored the interactions between titanium dioxide, silver, zinc oxide, carbon NPs, and silica dioxide NPs with gut microbiota [66,67]. AuNPs reduced the Firmicutes/Bacteroidetes ratio and the levels of probiotic *Lactobacillus*, supporting findings from studies involving silver NPs [67]. However, Zhu et al. reported that AuNPs could cause gut dysbiosis by altering the α-diversity, the Firmicutes/Bacteroidetes ratio, certain SCFAs-producing bacteria, and *Lactobacillus* in mice [68]. Thus, further detailed studies on the impact of AuNPs on the gut microbiome are necessary to explore the relationship between AuNPs and the host microbiome.

Utilizing 4,6-diamino-2-pyrimidinethiol-coated Au (D-Au) NPs, researchers aimed to combat bacterial infections and restore microflora balance. Mice were treated with varying doses (170, 1700, and 17,000 μg/kg) of D-Au and naked Au NPs for 28 days, with assessments including microflora composition, body weight, mouse biomarkers, and AuNPs distribution. Following the administration period, Au NPs were detected clustered or individually within intestinal epithelial cells. Overextended exposure, D-Au NPs showed no harm to intestinal cells and promoted the abundance of beneficial microbes, safeguarding intestinal microflora integrity without impacting the alpha diversity [69].

A study revealed a significant link between AuNPs gut microbiota and bone health, suggesting that AuNPs could effectively alleviate the severity of osteoarthritis induced by anterior cruciate ligament transection in mouse models through a mechanism dependent on the “microbiota-gut-joint” axis. AuNPs intervention led to changes in the abundance and diversity of gut microbiota associated with osteoarthritis, increased levels of SCFAs (acetic acid, propionic acid, butyric acid, isobutyric acid, and valeric acid), improved intestinal permeability, and balanced the dynamics of M1 and M2 macrophages. Subsequently, the reduced pro-inflammatory cytokine levels in the joints ultimately alleviated osteoarthritis severity. Moreover, the use of antibiotics to eliminate gut microbiota and fecal microbiota transplantation to improve the microflora further supported the involvement of a gut microbiota-dependent mechanism [70].

## 4. Gold Nanoparticles and Alzheimer’s Disease

Table 1 presents an overview of the various kinds of AuNPs used in AD diagnosis and management, classifying them into single, dual metal, hybrid, and functionalized AuNPs. Each category involves distinct modes of action and mechanisms employed against AD pathology. A comprehensive classification opens up multifaceted approaches that manipulate nanotechnology to address the challenges of AD, offering insight into potential therapeutic avenues in the field.

### 4.1. Single Gold Nanoparticles

NPs composed uniquely of gold atoms are considered as single AuNPs. Chiral nanoparticles could hinder Aβ_42_ aggregation and penetrate the BBB without apparent toxicity. Specifically, D3.3 exhibits enhanced binding affinity to Aβ_42_ and greater brain distribution compared to its enantiomer L3.3, resulting in more potent inhibition of Aβ_42_ fibrillation and improved restoration of behavioral deficits in mice models of AD. Coupling a small nanoparticle with chiral recognition elements presents a promising therapeutic strategy for AD [31].

**Figure 4:**
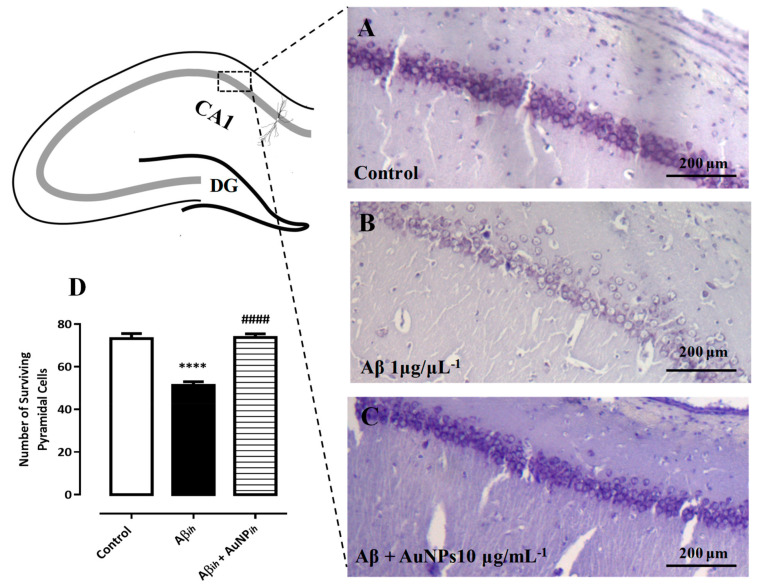
Variations in hippocampal pyramidal cells were observed across different conditions: (**A**) control, (**B**) Aβ_ih_, and (**C**) Aβ_ih_ + AuNP_ih_, with both qualitative (**A**–**C**) and quantitative (**D**) evaluations conducted. The presence of Aβih (1 μg/μL) resulted in changes in the integrity, linear shape, and cell count of pyramidal cells within the CA1 subfield of the hippocampus after 21 days. The treatment with AuNP_ih_ (10 μg/mL) notably prevented the detrimental effects induced by Aβ_ih_. Statistical analysis revealed significant differences (**** *p* < 0.0001) compared to the control group and (#### *p* < 0.01) compared to animals treated with Aβ_ih_ alone. CA1: Cornu Ammonis 1; DG: Dentate Gyrus; ih: Iliohypogastric nerve injections. The data is presented as mean ± SEM, with four samples in each group (Adapted with permission from Sanati et al., 2019 [32] and American Chemical Society).

The spatial learning and memory were assessed in a rat model of AD following intrahippocampal and intraperitoneal injections of AuNPs. The results showed that AuNPs could enhance the acquisition and retention of spatial learning and memory in Aβ-treated rats, as evidenced by a reduction in the time and distance required to find the hidden platform during training days and an increase in the time spent in the target quadrant during the probe test in the Morris water maze. Additionally, increased expressions of brain-derived neurotrophic factor (BDNF), cAMP response element binding protein, and stromal interaction molecules (STIM1 and STIM2) were observed, suggesting improved neural survival. Thioflavin S and Nissl staining of hippocampal slices confirmed the protective effect of AuNPs against Aβ fibrillation and its neurodegenerative consequences (Figure 4) [32].

The male Wistar rats were injected with okadaic acid (OA) to induce an AD model. Subsequent treatment with 20 nm AuNPs every 48 h for 21 days alleviated the effects of OA. OA increased the tau phosphorylation in the cortex and hippocampus, while AuNP treatment normalized it. Spatial memory impairment caused by OA was prevented by AuNP treatment. Neurotrophic factors (BDNF and nerve growth factor-β) decreased in the cortex and hippocampus due to OA, but AuNPs countered this effect. OA increased interleukin-1β levels in the hippocampus and cortex, while AuNPs mitigated it. OA and AuNP-treated groups exhibited elevated S100 levels in the cortex and hippocampus. However, AuNP treatment increased IL-4 in OA-treated animals. AuNPs countered OA-induced oxidative stress and preserved antioxidant status in the brain. Additionally, OA affected ATP synthase activity, but AuNPs maintained normal mitochondrial function. Long-term AuNP treatment prevented OA-induced neuroinflammation, mitochondrial dysfunction, and cognitive impairment, suggesting their potential as a promising treatment for AD [35].

The single AuNPs possess promising opportunities for addressing AD due to their unique properties for diagnostic and therapeutic applications. A berberine inhibition assay assessed the effectiveness of an AuNP dot blot immunoassay’s ability to detect Aβ_1–42_ production in SHG-44 cells. Co-culturing SHG-44 cells with varying concentrations of berberine over a duration resulted in a significant decrease in Aβ_1–42_ expression levels in the medium and cell lysate. The results suggest that the AuNP dot blot immunoassay is applicable for detecting Aβ_1–42_ in complex solutions and assessing inhibitors of Aβ_1–42_ production in cells. Furthermore, the study demonstrates the adaptability of the AuNP dot-blot immunoassay for analyzing practical samples, including the quantification of neurodegenerative disease biomarkers such as Aβ in conditions like AD, Huntington’s disease, and Parkinson’s disease [71]. Similarly, Kim et al. reported that AuNPs could detect the Aβ anti-aggregation properties of drug compounds in a short duration, making them a rapid tool for screening drugs targeting AD [72].

The aggregation of AuNPs induced by Aβ peptide causes a notable shift in the AuNPs’ surface plasmon resonance band, which makes it appropriate to screen for Aβ inhibitors. We could assess the ability of a molecule to hinder Aβ aggregation and compare the effectiveness of various inhibitors by measuring the absorption ratio at 600 and 520 nm. AuNPs-based assay offers a viable method for screening inhibitors against Aβ aggregation [73].

Aβ and α-synuclein fibrillation processes are major causes of AD. Different sizes and shapes of AuNPs exhibit varying inhibitory effects on fibrillation. Additionally, the concentration of AuNPs plays a role in affecting fibrillation. In detail, at higher concentrations (80 µg mL^−1^), AuNP shows stronger inhibitory effects than at low concentrations (5 µg mL^−1^), possibly due to providing more surface area for trapping Aβ monomers/oligomers, delaying fibrillation. Protein corona-coated AuNPs have weaker inhibitory effects, probably due to interactions between aggregation-prone regions of Aβ and the negative surface charge of the protein corona-coating, which may reduce affinity for binding. The attachment of Aβ’s 17–24 region to protein corona-coated AuNPs may be primarily mediated by lysine, affecting the inhibitory/acceleratory effects of AuNPs on Aβ fibrillation based on the composition of the protein corona [74].

The study by Chang et al. (2021) [75] reports the development of a novel graphene oxide (GO)-gold nanostar (GNS) construct on triangular electrodes for the sensitive and specific detection of microRNA-137 (miRNA-137), a reliable biomarker for AD. The sensor design involved modifying triangular electrodes with GO via an amine linker, followed by immobilization of complementary miRNA-137 conjugated to GNS. This strategy significantly enhanced probe density and hybridization efficiency. The device achieved a limit of detection of 10 femtomolar (fM), with sensitivity reaching 1 fM, and exhibited strong linearity. Importantly, the sensor showed high specificity, as control sequences (miRNA-21 and mismatched miRNA-137 variants) produced negligible current changes, while target miRNA-137 generated marked signal amplification. The inclusion of PEG-COOH further minimized nonspecific binding and improved performance. Compared to existing biosensing approaches for AD biomarkers, this platform demonstrated superior selectivity, stability, and anti-fouling properties. Overall, the study showed a low-cost, highly sensitive, and selective electrochemical biosensor for early AD detection, highlighting the diagnostic potential of nanomaterial-based microdevices in clinical applications [75].

The precipitation approach was employed to fabricate 50 nm-sized AuNPs. Cytotoxicity studies on PC12 cells revealed that Aβ_1–42_ combined with AuNPs exhibited decreased toxicity compared to untreated Aβ_1–42_ in terms of cell death, oxidative apoptosis, stress, and membrane leakage. It highlights the intricate interplay between NPs and protein oligomerization in neurodegenerative disorders, suggesting a potential avenue for novel pharmacological interventions targeting protein aggregation in AD. Further exploration into the physicochemical properties of AuNPs is warranted to refine the therapeutic properties of the developed AuNPs [76].

Recent advancements have introduced a novel class of chiral gold nanostructures synthesized using d- and l-cysteine–leucine dipeptides, which achieved a high chiral anisotropy factor (g-factor) of 0.1. These nanostructures were further organized into monolayer films (d-/l-Au monolayers) to enhance enantioselective detection capabilities. When employed as SERS substrates, these monolayers provided both molecular fingerprinting and chiral recognition in a single spectrum, enabling label-free discrimination of d- and l-kynurenine (Kyn). The enantioselectivity was attributed to differing adsorption energies on the crystal lattice plane. Sensitivity tests revealed that the l-Au monolayer could detect l-Kyn down to 3.7 nM, while the d-Au monolayer achieved a detection limit of 3.6 nM for d-Kyn. Importantly, elevated levels of d-Kyn were observed in serum samples from AD patients compared to healthy controls, a distinction not seen with l-Kyn, suggesting d-Kyn as a promising new biomarker for early AD diagnosis. This represents a significant step forward in chiral biomolecule detection and its application in clinical diagnostics [93].

A recent study explored the therapeutic potential of afzelin, a natural compound derived from *Ribes fasciculatum*, when conjugated with AuNPs to improve its efficacy and address challenges related to low bioavailability. The combination, referred to as AuNP–afzelin, was hypothesized to exhibit superior neuroprotective effects compared to afzelin alone. In a scopolamine-induced AD mouse model, central administration of AuNP–afzelin (equivalent to 10 ng of afzelin) significantly improved cognitive performance and memory function, surpassing the outcomes of higher doses of afzelin administered independently. Further analysis revealed that AuNP–afzelin enhanced neuronal survival, restored cholinergic activity, and activated key neuroprotective signaling pathways, including BDNF-pCREB-pAkt, along with increased hippocampal expression of doublecortin, a marker of neurogenesis. These findings suggest that gold nanoparticle-based delivery of afzelin represents a promising therapeutic strategy for alleviating cognitive deficits in neurodegenerative diseases and warrants further investigation for drug development [94].

Single AuNPs continue to demonstrate significant promise as versatile tools in the diagnosis and treatment of AD. Their unique physicochemical properties enable sensitive detection of key biomarkers, efficient inhibition of pathological protein aggregation, and neuroprotective effects that improve cognitive function in experimental models. Additionally, their adaptability in functionalization and biocompatibility opens new pathways for targeted drug delivery and therapeutic intervention. As research advances, single AuNPs stand out as a powerful platform with the potential to bridge gaps in early diagnosis and effective treatment, offering hope for improved management of neurodegenerative disorders.

### 4.2. Dual Metal Gold Nanoparticles

The NPs composed of gold and other metals are called dual-metal AuNPs. The dual metal NPs typically consist of a core made of gold surrounded by a shield or composite containing one or more additional metals. The combination of gold with other metals reveals unique properties of the NPs, such as enhanced catalytic activity, improved stability, and tunable optical and electronic properties.

Au@Pt/AuNPs were synthesized through a chemical process involving successive galvanic replacement reactions and metal depositions from initial AuNPs. The protruding Au growth on Au@Pt NPs facilitated easy bioconjugation with antibodies, as the high catalytic Pt surface enabled sensitive recognition via electro-catalyzed water oxidation reaction (WOR) at neutral pH. Additionally, the synergy between Au and Pt metals on the NP surface enhanced catalytic activity and improved the detection sensitivity. Cyclic voltammetry and chronoamperometry evaluated Au@Pt/Au NPs’ electrocatalytic action toward WOR. The chronoamperometric current, noted at a fixed potential of +1.35 V, served as the analytical indicator, admitting quantification of Au@Pt/Au NPs at 1013 NPs/mL levels. The improved electrocatalytic method quantified the conformationally modified p53 peptide, an AD biomarker, in a competitive immune assay using magnetic bead (MB) platforms, detecting levels as low as 66 nM. The system’s performance was validated by analyzing plasma samples from cognitively healthy subjects in a real scenario as novel tags for determining AD biomarkers [77].

The co-incubation of Aβ with SH-SY5Y cells reduced cell survival to 37% compared to the control group. However, when exposed to concave cubic or concave cubic quercetin-modified Au-palladium NPs (P-80@AuPd or Qu), cell survival rates were increased to 68 and 74%, respectively. Notably, concave cubic Qu@P-80@AuPd significantly attenuated Aβ-mediated cytotoxicity, with cell survival rates reaching nearly 90% at a concentration of 10 µg/mL. The result suggests that concave cubic Qu@P80@AuPd effectively shields SH-SY5Y cells from Aβ-induced cytotoxicity by enhancing intracellular Aβ clearance. Activation of caspase-3, a pivotal factor in promoting apoptosis, was analyzed. Caspase-3 activity increased to 218% when SH-SY5Y cells were exposed to Aβ, compared to the control group (100%). However, concave cubic Qu@P80@AuPd significantly inhibited caspase-3 activity in SH-SY5Y cells, thereby reducing apoptosis, compared to concave cubic P-80@AuPd or Qu [78].

The integration of gold with other metals to create dual-metal nanoparticles has opened new avenues in the diagnosis and treatment of AD. These hybrid structures enhance the functional capabilities of conventional gold nanoparticles by improving sensitivity, selectivity, and therapeutic potential. Whether in biosensing platforms for early biomarker detection or as targeted therapeutic agents that modulate Aβ toxicity, dual-metal AuNPs have shown significant promise. Their ability to penetrate biological barriers, improve bioavailability, and facilitate precision diagnostics supports their growing relevance in translational medicine. As research continues to evolve, dual-metal AuNPs stand at the forefront of nanomedicine, offering innovative solutions to address the complex pathology of AD and bringing us closer to effective clinical interventions.

### 4.3. Hybrid Gold Nanoparticles

Hybrid AuNPs are a class of NPs that blend gold with other materials, such as biomolecules, organic polymers, and inorganic materials. These hybrid NPs possess synergistic properties derived from the combination of Au with the secondary material, offering unique advantages for various biomedical applications. Utilizing the potential of hybrid AuNPs represents a novel strategy for addressing AD by combining the unique properties of gold with tailored functionalities, paving the way for innovative therapeutic and diagnostic approaches.

In AD, Aβ_1–42_ levels in plasma and cerebrospinal fluid (CSF) are reduced due to aggregation in the brain. Studies showed that the Aβ_1–42_ plasma levels of 18.05 ± 5.1 pg/mL for Alzheimer’s patients and 27.11 ± 2.45 pg/mL for older normal controls. A cohort experiment on healthy subjects across different age ranges revealed a normal plasma Aβ_1–42_ level of 17.65 ± 5.71 pg/mL. Furthermore, Aβ_1–42_ levels in CSF were 1678 ± 436 pg/mL for healthy individuals and 709 ± 304 pg/mL for Alzheimer’s patients [79]. These Aβ_1–42_ levels fall inside the linear range of detection for the biosensor, indicating its capability to accurately determine Aβ_1–42_ levels as low as those reported in Alzheimer’s patients’ biological fluids [79,95,96,97].

To ensure the clinical applicability of Au-based NPs, it is crucial to assess the performance of a biosensor in physical (real) samples rather than buffer solutions without compromising its effectiveness. In this context, an established method was employed to detect Aβ_1–42_ in CSF using an immunosensor based on anti-Aβ-AuNP/nickel ferrite coated graphene oxide-chitosan nanocomposite (Au/NiFe_2_O_4_@GO-Ch/GCE). Functional evaluation was conducted by testing the immunosensor’s ability to detect different known concentrations of Aβ_1–42_ in CSF samples. The differential pulse voltammetry results confirmed that the immunosensor selectively detected Aβ_1–42_. The developed immunosensor can be used in a clinical setting to detect Aβ_1–42_ in CSF samples [80].

Three different probe surfaces were compared for the identification of Aβ oligomers (AβO): (1) Au-urchin coupled antibody, (2) antibody-aptamer involves two steps, and (3) a dual probe alongside antibody and aptamer, involving a single step. Firstly, the Au-urchin antibody was bound to a nano horn-altered area via an amine (3-aminopropyl) triethoxysilane (APTMS) linker, followed by detecting AβO. In the second experiment, Au-urchin antibodies were immobilized; first, an aptamer was included until saturation, and AβO was detected using the thiol-linked aptamer. In the last experiment, the aptamer was bound to the persistent Au-urchin before the immobilization of the antibody, and AβO detection was compared with previous methods. The dual probe-modified surface, with aptamer-antibody-altered Au-urchin bind to nano horns, showed improved AβO recognition with a detection limit of 10 fM. Enhanced current changes were observed due to the dual probe interacting with more AβOs. Control experiments confirmed specific detection of AβO, and spiking AβO into artificial CSF did not interfere with its detection, indicating selective determination of AβO. This dual-probe-modified electrode surface offers the potential for identifying AβO and diagnosing AD [81].

The inhibition of Aβ fibrillation by the L-glutathione-coated AuNPs and nanoclusters (AuNCs) has been reported. The larger AuNPs (AuNPs1 and AuNPs2) accelerated the growth of Aβ fibrils, while the smaller AuNPs (AuNPs3) inhibited the growth of Aβ fibrils. AuNCs significantly prolonged the growth phase of the fibrillation curves, resulting in a sharp decrease in fluorescent intensity with increasing concentration. AuNCs effectively inhibited the further aggregation of Aβ and the formation of larger oligomers, such as micelles and protofibrils [82].

**Figure 5:**
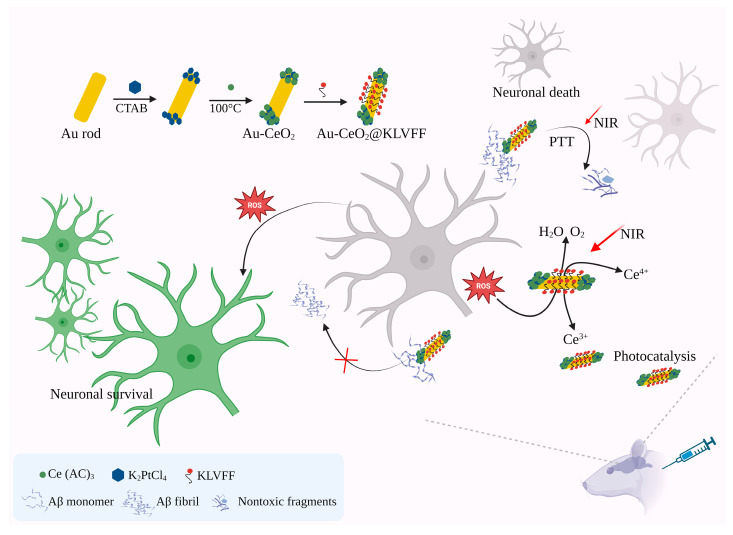
In conjunction with the semiconductor cerium oxide, gold nanorods exhibit antioxidant stress and inhibition of Aβ via photocatalysis and the photothermal effect, offering a multimodal approach to AD therapy. The figure has been recreated with permission from Ge et al., 2022 [83] and the American Chemical Society. It was created using BioRender.com. Au-CeO_2_: Gold-cerium oxide; PTT: Photothermal Therapy; NIR: Near-Infrared region; CTAB: Cetyltrimethylammonium bromide; Ce (AC)_3_): Cerium acetate; K_2_PtCl_4_: Potassium tetrachloropropionate; KLVFF: Pentapeptide fragments (Aβ_16–20_).

The successful creation of pentapeptide fragments (KLVFF) KLVFF@Au-CeO_2_ nanocomposites marks a significant advancement in multimodal therapy for AD. This novel structure, with distinct spatial separation, notably enhances the effectiveness of photothermal conversion and catalytic function. Moreover, by incorporating KLVFF targeting peptides and leveraging photothermal-induced BBB opening, these nanocomposites enhance drug concentrations effectively via active and passive targeting mechanisms. Importantly, nanocomposites demonstrate exceptional biocompatibility and stability. Animal studies indicate that KLVFF@Au-CeO_2_ effectively improves cognitive function in AD mice by addressing multiple aspects of AD pathology, including inhibiting Aβ monomer aggregation, breaking down Aβ fibers, and scavenging ROS. Integrating effective photocatalysis and multitarget synergism represents a promising new direction for leveraging nanotechnology in AD therapy. Figure 5 demonstrates that the photothermal effect induced by near-infrared irradiation enhances BBB permeability while the KLVFF-targeted function mitigates damage to normal tissue. The interactive action of photothermal decomposition of the Aβ polymer, antioxidant stress, and inhibition of Aβ monomer aggregation protects nerve cells [83].

Identifying effective inhibitors of Aβ aggregation is a key step in the development of therapeutics for AD. Conventional biochemical and cellular screening methods often fall short when applied to high-throughput screening of natural products. To address this, an AuNPs-based assay was utilized to rapidly screen marine-derived fungal crude extracts and purified compounds for anti-Aβ aggregation activity. Several extracts, including DLS2008001(M), BM3T2(M), DLEN2008005(M), TBG1-16(P), and TBG1-13(P), demonstrated inhibitory effects comparable to human serum albumin, the positive control, at 500 μg/mL. Among the purified compounds, nidulin, aspergillusidone G, and butyrolactone I showed notable activity in preventing Aβ aggregation. Transmission electron microscopy (TEM) further validated the anti-aggregation effects of selected extracts, especially TBG1-16(P), DLS2008001(M), and BM3T2(M), by visualizing reduced Aβ fibril formation. Additionally, molecular docking analysis confirmed strong interactions and favorable binding affinities between the active compounds and multiple Aβ peptide isoforms. Overall, this study highlights the efficiency of the GNP-based screening approach and underscores marine fungi as a promising reservoir of small-molecule Aβ aggregation inhibitors for AD research [98].

The integration of AuNPs with diverse functional and structural materials has ushered in a new era of hybrid nanoplatforms for AD diagnosis and therapy. These hybrid AuNPs exhibit synergistic properties that go beyond the capabilities of gold alone, enabling enhanced detection of Aβ species, inhibition of fibril formation, modulation of oxidative stress, and effective brain targeting. From advanced biosensors capable of detecting minute concentrations of Aβ peptides to multifunctional nanocomposites that combine photothermal therapy with catalytic activity, hybrid AuNPs offer tailored, multifaceted solutions for tackling the complex pathology of AD. With promising results in both in vitro and in vivo models, these nanostructures hold strong potential for translation into clinical applications, paving the way for earlier diagnosis, targeted intervention, and improved outcomes in neurodegenerative disease management.

### 4.4. Functionalized Gold Nanoparticles

The chemically modified gold or AuNPs coated with various functional groups/molecules have been considered functionalized AuNPs. Integrating functionalized AuNPs represents a cutting-edge approach in AD research, offering precise targeting and enhanced therapeutic potential through tailored surface modifications. A study assessed the impact of CLPFFD peptide-functionalized hollow Au nanospheres and Au nanorods on Aβ fibril formation. The results indicated that no significant reduction in fibril formation was observed after near-infrared irradiation, indicating that irradiation alone does not affect Aβ fibril growth. TEM analysis of non-irradiated and irradiated samples of Aβ fibrils incubated with AuNP conjugates revealed less fibril formation compared to pure Aβ fibrils, suggesting a potential inhibitory effect of the AuNP conjugates on fibril formation [84].

*Heliotropium eichwaldi*-functionalized AuNPs (HE-AuNPs) demonstrated remarkable anti-acetylcholinesterase (AChE) activity, with an inhibition rate of 67% and an IC_50_ value of 88 μg. At the maximum concentration tested (150 μg), pure gold salt inhibited AChE by 33%, whereas HE-AuNPs inhibited it by 93%. Lineweaver–Burk plot indicated that HE-AuNPs inhibit AChE noncompetitively, with unchanged *K_m_* but decreased *V_max_* from 20 to 6.24. HE-AuNPs did not affect *K_lapp_* but increased *V_maxiapp_* from 17.5 to 29. *K_m_*, *K_l_*, and *K_i_* values were 0.041 mM, 66 μg, and 25 μg, respectively. Overall, the results highlight the potential of HE-AuNPs as anti-AChE agents, indicating their suitability for enhancing drugs for AD treatment, facilitated by their green synthesis methodology [85].

AuNPs surface-functionalized with mimosine (Mimo-AuNPs) were investigated for their impact on the Aβ fibrillization process, toxicity, and ability to cross the BBB. The study found that Mimo-AuNPs effectively suppressed spontaneous and seed-induced aggregation of Aβ_1–42_, as evidenced by thioflavin T kinetic assays, fluorescence imaging, and electron microscopy. Spectroscopic studies, molecular docking, and biochemical analyses indicated that Mimo-AuNPs interacted with the hydrophobic domain of Aβ_1–42_, specifically from Lys16 to Ala21, stabilizing the peptide in its monomeric state and preventing its conformational shift toward β-sheet structures. Moreover, Mimo-AuNPs induced the disassembly of mature Aβ_1–42_ fibers and increased neuronal viability by reducing tau protein phosphorylation and oxyradical production. Overall, the findings suggest that Mimo-AuNPs hold promise in attenuating Aβ fibrillization and neuronal toxicity in AD [86].

AuNPs functionalized with polypeptides (PFGNP) were synthesized with polypeptides Cys-Gly-Gly-Gly-Leu-Pro-Phe-Phe-Asp and Cys-Gly-Gly-Gly-Gly-Gly-His onto AuNPs through gold-sulfur bonds. The bifunctional nanoparticles effectively chelate copper ions and simultaneously inhibit Aβ_1–42_ fibril formation. Moreover, PFGNP demonstrates notable protection against Aβ-induced cytotoxicity in SH-SY5Y cells. Given the favorable biocompatibility and non-toxic nature of AuNPs and peptide molecules, the findings suggest that PFGNP could be a potent therapeutic agent for AD [87].

Recent breakthroughs in employing AuNPs against AD exhibit their growing significance as a resourceful and effective tool in combating the complexities of AD. The bilayer immunoassay employs magnetic beads with modified antibodies to capture ADAM10 and AuNPs as electrochemical labels. The assay accurately detects ADAM10 in diluted plasma, with a LOD of 32.5 pg/mL and a dynamic linear range of 10.0–1000.0 pg/mL. Evaluation with 23 plasma samples from elderly individuals, including those with AD, mild cognitive impairment, and healthy controls, demonstrates the correlation between ADAM10 levels and disease progression. The study claimed that the magnetoimmunoassay could be a rapid and cost-effective serological test for AD, targeting ADAM10 biomarkers. Detection in plasma offers safety and ease of collection compared to CSF. Combining the magneto-immunoassay with screen-printed electrodes enhances its point-of-care applications compared to traditional ELISA methods. Thus, the device presents a promising alternative for early diagnosis and monitoring of AD progression [88].

The treatment for neurodegenerative diseases such as AD remains formidable, primarily due to the brain’s limited capacity for neuroregeneration. Recent studies investigate the potential of external magnetic field (MF) stimulation combined with nerve growth factor functionalized superparamagnetic iron oxide-gold NPs (NGF-SPIO-Au NPs) to induce calcium influx, membrane depolarization, and enhance neuronal differentiation. It demonstrates that dynamic MF (DMF) stimulation, particularly at a frequency of 1 Hz, significantly improves total intracellular calcium influx in PC-12 cells by 300% and 535% when combined with NGF-SPIO-Au NPs compared to DMF alone and static MF (SMF) with NPs, respectively. The enhanced calcium influx is attributed to successive membrane depolarization facilitated by DMF [89]. Furthermore, cellular uptake experiments utilizing sodium azide confirm that DMF promotes the cellular uptake of NGF-SPIO-Au NPs via endocytosis. Notably, DMF also upregulates neural differentiation markers like β3-tubulin and cell adhesive molecules such as integrin-β1 in the presence of NGF-SPIO-Au NPs, while SMF fails to produce similar effects. It points out the potential of noninvasive DMF-stimulated NPs in regulating intracellular calcium influx and promoting neuronal differentiation and neuroregeneration [89].

The BBB-on-a-Chip (BBB-oC) model, which incorporates pericytes, endothelial cells, and human astrocytes, coupled with a trans-endothelial electrical resistance (TEER) measuring system, mimics the neurovascular system of the brain. The study introduced a groundbreaking nanotherapeutic agent, GNR-PEG-Ang2/D_1_, comprising gold nanorods decorated with polyethylene glycol and biofunctionalized through peptides, including Angiopep-2. The formulation was specifically engineered to traverse the BBB and target the underlying pathology of AD by inhibiting Aβ fibrillation, a hallmark of the disease. Importantly, the study determined the non-cytotoxic limit of GNR-PEG-Ang2/D_1_ and demonstrated its harmless effect on the neurovascular system. Permeability analyses exemplified that Ang2 augments the permeability of GNR-PEG-Ang2/D_1_, with endocytosis identified as a potential entry mechanism. It highlights the utility of the TEER-BBB-oC system as a valuable tool for the rapid and cost-effective evaluation of drug permeability across the BBB. Furthermore, this approach provides a platform to assess the impact of nanotherapeutic agents on the CNS, potentially paving the way for innovative treatments and initiating the drug development process for neurodegenerative diseases [90].

**Figure 6:**
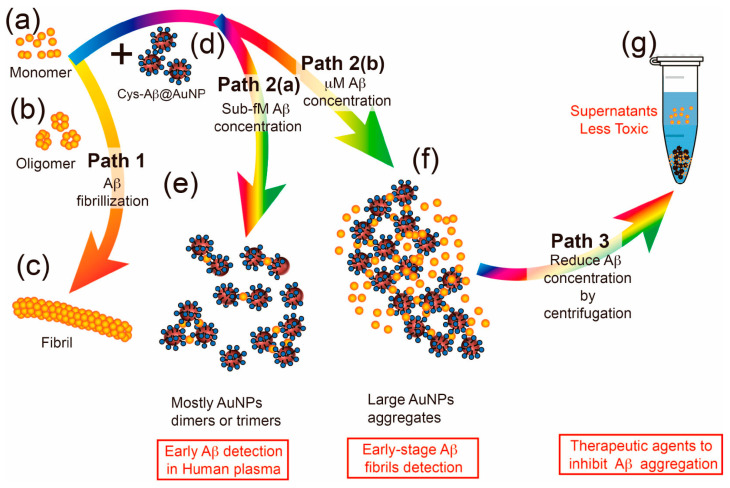
A diagrammatic depiction outlines three proposed pathways. Path 1 shows the sequential formation of Aβ (**a**) monomers progressing to (**b**) oligomers and ultimately (**c**) fibrils. Introducing (**d**) Cys-Aβ@AuNPs redirects the normal aggregation pathway to either path 2a or 2b, depending on the concentration of Aβ monomers, with sub-femtomolar or micromolar concentrations leading to these pathways, respectively. Path 2(a) predominantly yields AuNP dimers or trimers (**e**) in solution, resulting in significant extinction variations. At the same time, path 2(b) leads to the formation of large AuNP aggregates (**f**), facilitating the detection of early-stage Aβ oligomerization. Pathway 3 demonstrates that the supernatant post-centrifugation (**g**) exhibits reduced aggregation propensity due to the decreased Aβ concentration (Adapted with permission from Chang et al., 2024 [91] and the American Chemical Society).

A recent study reveals a highly sensitive technique for detecting minuscule amounts of Aβ peptide in a solution, which is crucial for identifying the early stages of Aβ aggregation. Figure 6 illustrates the concept of the method. Path 1 illustrates the conventional route of Aβ aggregation, where monomers progress to oligomers and eventually fibrils. Introducing cysteine-Aβ peptide-conjugated AuNP (Cys-Aβ@AuNP) alters this path, leading to alternative routes based on Aβ monomer concentration. When exposed to sub-femtomolar Aβ monomers (Path 2a), most Cys-Aβ@AuNP molecules interact with Aβ monomers to form AuNP dimers or trimers, causing noticeable changes in extinction due to surface plasmon resonance coupling between AuNPs [91].

Conversely, exposure to micromolar Aβ monomers (Path 2b) results in large-scale AuNP aggregates, reflected in a single broad extinction peak. Additionally, the proposed Cys-Aβ@AuNP holds promise for developing effective therapeutic agents to impede Aβ aggregation. The results are expected to lead to innovative approaches in early detection and inhibition of Aβ aggregation [91].

Upon incubation with Aβ peptides, the Cys-Aβ@AuNP aggregates change their absorption spectra, indicating an interaction. This interaction enables the identification of early-stage Aβ oligomerization, which is not detectable by conventional methods, such as Thioflavin T fluorescence. Additionally, the binding of Aβ peptides by Cys-Aβ@AuNP effectively inhibits the formation of toxic Aβ oligomers or fibrils. Therefore, Cys-Aβ@AuNP can be utilized to detect sub-femtomolar Aβ peptides and oligomerization, while also reducing Aβ concentration and inhibiting aggregation, offering potential therapeutic applications in AD and related neurodegenerative disorders [91].

The aqueous AuNPs and *Acorus calamus* extract showed stronger antioxidant effects than the standard drug rivastigmine. Aqueous AuNPs demonstrated particularly high antioxidant activity, surpassing both *A. calamus* extract and rivastigmine in terms of effectiveness. This suggests that AuNPs can penetrate the BBB, increase acetylcholinesterase levels, and simultaneously reduce oxidative stress. The results indicate that AuNPs could be a promising candidate for developing anti-AD drugs [92].

Wu et al. (2020) [99] report the successful synthesis of chiral AuNPs coated with d/l-penicillamine. These chiral ligands impart distinct biological properties to the nanoparticles while maintaining good stability and dispersibility. The functionalized AuNPs were tested in PC12 neuronal cells to evaluate their potential in AD therapy. Results showed that both AuNPs exhibited protective effects against Aβ-induced cytotoxicity, with notable differences depending on the chirality. The chiral coating influenced cell viability, oxidative stress levels, and cellular uptake, suggesting that stereochemistry plays a critical role in modulating NPs-cell interactions. Importantly, the findings highlight that d-penicillamine-coated AuNPs provided stronger neuroprotection compared to the l-form, reducing oxidative damage more effectively and preserving neuronal function [99].

A recent study explored the eco-friendly synthesis of AuNPs using natural phytochemicals morin hydrate, polydatin, and berberine chloride to address BBB permeability. In oxidative stress models using paraquat-treated SH-SY5Y neuroblastoma cells, the nanoparticles showed enhanced ROS scavenging, superior antioxidant activity, and higher biocompatibility compared to both conventional AuNPs and their parent phytochemicals. Among the three, polydatin-conjugated AuNPs were particularly effective, reducing cellular ROS and lipid peroxidation to near-normal levels. These findings highlight the promise of phytochemical-based, green-synthesized AuNPs as potential therapeutic agents for oxidative stress-associated neurodegenerative conditions, offering a novel strategy that combines natural antioxidants with nanotechnology to improve treatment outcomes in central nervous system disorders [100].

The development of functionalized AuNPs marks a significant advancement in AD therapeutics, enabling targeted intervention at the molecular level. Through strategic surface modifications ranging from peptides and polypeptides to small molecules like mimosine, these engineered nanoparticles demonstrate enhanced capabilities in inhibiting Aβ aggregation, disrupting existing fibrils, and reducing neurotoxicity. The multifunctionality of these platforms, including their ability to cross the BBB and modulate oxidative stress, underscores their potential as versatile and biocompatible tools for combating AD pathology. Continued exploration of functionalized AuNPs holds great promise for translating nanotechnology-based solutions into effective clinical therapies for neurodegenerative diseases.

Collectively, these recent advancements underscore the multifaceted potential of gold nanoparticles in the diagnosis, monitoring, and treatment of AD. From enhancing biomarker detection and crossing the BBB to mitigating oxidative stress and inhibiting Aβ aggregation, AuNPs demonstrate exceptional versatility and promise. Innovations such as biofunctionalized nanorods, magneto-responsive nanocomposites, and phytochemical-based green synthesis are opening new avenues for early intervention and targeted therapy in neurodegenerative disorders. As research continues to evolve, AuNPs-based platforms may emerge as pivotal tools in addressing the complex pathophysiology of AD, offering hope for more effective and accessible solutions in the fight against this debilitating disease.

## 5. Clinical Applications of Gold Nanoparticles

The expanding display of clinical applications for AuNPs highlights their applications in various fields, including targeted drug delivery, imaging, and diagnostics. Despite this, clinical trials for AD nanomedicines have been inadequate, underscoring the need for further research to bridge the gaps in the field. While some nanomedicines have been successful in clinical applications, the quest for effective treatments continues [101].

Table 2 presents recent clinical trials involving the use of AuNPs and other nanoparticles in the treatment of AD and other neurodegenerative conditions. The clinical trials encompass various phases of research, ranging from recruitment to completion. These trials highlight the increasing interest in utilizing nanotechnology to address the complex pathology of neurodegenerative diseases, potentially opening up avenues for innovative therapeutic approaches. Their additional objective was to explore the potential of nanoparticles for diagnosing and clinically managing neurodegenerative disorders. Despite potential findings from these clinical investigations not meeting expected standards or being disclosed, ongoing efforts in clinical research persist unchanged [102,103].

## 6. Translocation of AuNPs Across the Blood–Brain Barrier

AuNPs employ several strategies to overcome the BBB following systemic administration. Receptor-mediated transcytosis is one of the most widely studied mechanisms. For example, transferrin-functionalized AuNPs (sized 45 and 80 nm) were shown to successfully cross the BBB and reach the brain parenchyma in mice, with brain accumulation confirmed by inductively coupled plasma mass spectrometry and electron microscopy. Importantly, the study revealed that intermediate binding affinity to the transferrin receptor maximized transport while avoiding endothelial trapping [112]. Similarly, Angiopep-2-modified Au nanorods not only crossed the BBB but also improved barrier function. In a BBB-on-a-chip model, permeability assays demonstrated nanoparticle passage, while trans-endothelial electrical resistance increased significantly over time. Confocal microscopy further showed enhanced expression of tight junction proteins zonula occludens-1 and vascular endothelial cadherin, indicating that these nanoparticles could both penetrate the barrier and strengthen its integrity [90].

Other mechanisms include adsorptive-mediated transcytosis using cationic surfaces, “Trojan horse” delivery via monocytes or macrophages that naturally traverse the BBB, and transient barrier opening through focused ultrasound or near-infrared photothermal stimulation. In parallel, intranasal (nose-to-brain) delivery has emerged as an effective approach to bypass the BBB altogether by exploiting the olfactory and trigeminal nerve pathways. For example, intranasal administration of Au nanorods in mice demonstrated brain uptake within minutes [113]. Recently, apolipoprotein E-mimetic peptide-functionalized AuNPs administered intranasally demonstrated enhanced brain targeting in an ischemia model, improving neuronal survival and cognitive outcomes, which highlights the translational potential of functionalized AuNPs for AD therapy [114].

Together, these results demonstrate that AuNPs can overcome the BBB by multiple systemic strategies, including receptor-mediated, adsorptive-mediated, Trojan horse, and transient disruption routes, and, importantly, can also bypass the BBB entirely through intranasal delivery. These complementary approaches not only improve drug delivery efficiency but also open new opportunities for targeted diagnosis and therapy in AD.

## 7. Safety and Toxicity of AuNPs

AuNPs have promising biomedical applications, but a detailed evaluation of their toxicological implications, especially neurotoxicity, tissue accumulation, and long-term safety, is essential. Toxicity is highly dependent on several factors, including size, shape, coating, etc. [115,116,117,118,119].

The small AuNPs demonstrate increased cytotoxicity in vitro and in vivo relative to larger counterparts, due to enhanced cell entry, reactive surface area, and possible DNA interaction [115]. Furthermore, ultra-small particles (<4 to 5 nm) could bind within DNA grooves, raising concerns about genotoxicity, transcriptional interference, and epigenetic disruption [118,120].

Surface properties and coating significantly modulate the toxicity of NPs, especially AuNPs with stabilizing ligands such as bovine serum albumin (BSA) or polyethylene glycol (PEG) often exhibit reduced acute toxicity, whereas particles stabilized with surfactants like cetyltrimethylammonium bromide demonstrate pronounced cytotoxicity due to free ligand-mediated damage [118,121].

Dose- and time-dependent effects reveal that while low doses may be tolerated, higher or repeated exposure risks accumulation and inflammation. In vivo studies of BSA-coated AuNPs over 120 days showed persistent accumulation in the liver, spleen, and kidneys, along with increased expression of inflammatory mediators, slight organ enlargement, and early fibrotic changes that highlighting potential chronic toxicity even after a single administration [117]. Similarly, green-synthesized ultra-small AuNPs (~7.8 nm) administered to rats at low doses (10 mg/kg) showed minimal acute toxicity, but higher doses led to detectable accumulation in brain glial cells, underlining dose-dependent biodistribution concerns [122].

Bioaccumulation and long-term redistribution patterns further complicate safety profiles. GSH- versus BSA-coated nanoclusters exhibit distinct clearance dynamics: GSH-protected clusters clear more efficiently via the kidneys, with significantly less hepatic or splenic retention and reversible acute toxicity, while BSA-coated clusters accumulate sharply and persist in organs, driving prolonged inflammatory responses [117,123]. Glutathione-protected gold nanoclusters demonstrated unexpected V-shaped biodistribution, with initial clearance followed by redistribution from muscle, leading to reaccumulation and sustained liver toxicity up to 90 days post-injection [124].

Neurotoxicity remains a concern; for instance, AuNP exposure triggered astrogliosis, increased seizure activity, and other neural perturbations, suggesting possible neuroinflammatory or excitotoxic effects, though these appear sensitively dependent on particle characteristics and administration protocols [125]. Some studies show that colloidal AuNPs can cross the BBB and accumulate in neurons, but clear toxicity is not always seen, highlighting the need for more detailed mechanistic studies [118,119,121].

## 8. Future Perspectives

Exploring the current limitations and future perspectives of AuNP-based interventions for AD offers valuable insights into refining strategies and unlocking their full therapeutic potential in clinical applications. Current clinical therapy for AD primarily aims to manage symptoms and provide supplementary brain nutrition, offering comfort care without addressing the underlying cause of the disease [101]. The complications of AD pathology and the difficulties in delivering drugs beyond the BBB are the major limitations to managing AD effectively. Nanocarriers have garnered attention owing to their exceptional biocompatibility, stability, degradability, flexible drug-loading methods, safety, surface modifiability, and controllable drug release. They are interesting candidates for directed treatment for AD and other CNS diseases. However, commercial production of nanomedicine remains a significant challenge. The BBB, comprising pericytes, astrocytes, capillary basement membrane, and endothelial cells, acts as a protective barrier, limiting the entry of definite molecules or pathogens from the circulatory system into the brain [126]. The capability of nanomedicine in treating neurodegenerative diseases is immense. Although some nanomedicines are undergoing clinical trials for AD, none have been approved yet. Most nanomedicine research is currently in the preclinical stage [127].

The research must prioritize overcoming BBB-related barriers and enhancing drug delivery systems, thereby advancing the field of nanomedicine and facilitating the development of more efficient treatments for AD and other CNS diseases [101]. Further investigation into the physicochemical properties of AuNPs is warranted to deepen our understanding and potentially leverage their effects for therapeutic purposes [76]. Additionally, the limited understanding of the physiological and pathological mechanisms underlying AD poses a challenge to developing efficient nanomaterial-based treatments. If the fundamental biological mechanism of AD is revealed, nanomaterial-based treatments are expected to play an increasingly significant role in its treatment [101].

## 9. Conclusions

AuNPs represent a versatile nanoplatform with the potential to transform both the diagnosis and treatment of AD. Their tunable physicochemical properties, ease of functionalization, and intrinsic optical features enable applications ranging from inhibition of Aβ aggregation to ultrasensitive detection of AD biomarkers. Importantly, the diversity of AuNP designs, such as ultrasmall glutathione-protected nanoclusters with improved renal clearance, chiral nanostructures with enhanced optical selectivity, and hybrid or bimetallic Au-based nanozymes with catalytic and multimodal imaging functions, demonstrates how tailored modifications can directly address major challenges such as BBB penetration, bioaccumulation, off-target toxicity, and diagnostic reliability. By integrating these advanced AuNP variants with smart delivery strategies and thorough safety evaluations, it is possible to move beyond proof-of-concept studies toward clinically relevant theranostic systems. Thus, while limitations remain, the structural and functional adaptability of AuNPs provides a promising pathway to overcome current barriers in AD management and accelerate the translation of nanotechnology into effective patient care. Further research is crucial for elucidating the mechanisms of action and optimizing the physicochemical properties of AuNPs, thereby realizing their therapeutic potential in addressing AD and other CNS disorders.

## Figures and Tables

**Table 1:** Gold nanoparticles (AuNPs) in the diagnosis and management of Alzheimer’s disease.

Nanoparticle	Classification	Model, Dose, and Route of Administration	Neurological Application	Observations	Study
Chiral AuNPs	Single metal NPs	In vitro and AD mice model; 25 mg/kg; Intravenous.	Inhibition of Aβ42 aggregation	Prevents Aβ aggregation by adsorbing Aβ monomers.	[31]
AuNPs	Single metal NPs	AD rat model; 1 or 10 or 100, or 200 µg/mL; Intrahippocampal and intraperitoneal.	Spatial memory impairment and neural loss	Alleviates spatial memory impairment.	[32]
AuNPs	Single metal NPs	AD rat model; 100 µg; Intracerebroventricular.	Spatial memory deficit prevention	Normalize the tau phosphorylation. Prevents spatial memory deficits. AuNPs activate antioxidant defense mechanisms. Preserves normal mitochondrial function.	[35]
AuNPs	Single metal NPs	In vitro	Detection of Aβ peptide	Detection of Aβ peptide	[71]
AuNPs	Single metal NPs	In vitro	Detection of Aβ peptide	Facilitates the discovery of efficient drug components for the treatment of AD.	[72]
Label-free AuNPs	Single metal NPs	In vitro	Detection of Aβ inhibitors	Effective and rapid screening of Aβ inhibitors.	[73]
AuNPs	Single metal NPs	In vivo *	Aβ fibrillation process	Prevents the hot spot regions of Aβ monomers from binding to each other and effectively blocks the fibrillation process.	[74]
Graphene oxide-gold nanostar	Single metal NPs	In vitro	Detection of miRNA-137 for early diagnosis of AD	It enables specific detection of miRNA-137, achieving a detection limit of 10 fM and a sensitivity of 1 fM, crucial for early AD diagnosis.	[75]
AuNPs	Single metal NPs	In vitro	Development of cytotoxic oligomers by binding to proteins in AD	AuNPs with Aβ_1–42_ oligomers caused a stronger increase in caspase-3 activity compared to control oligomers.	[76]
Au@Pt/Au core@shell NPs	Dual metal NPs	In vitro	Quantification of p53 peptide as a biomarker of AD	Aids in quantifying the altered p53 peptide.	[77]
Quercetin-modified gold-palladium nanoparticles	Dual metal NPs	In vitro	As a potential autophagy inducer for the treatment of AD	Concave cubic Qu@P-80@AuPd influences the autophagy in SH-SY5Y cells in a dose-dependent manner.	[78]
Microporous gold nanostructure	Hybrid AuNPs	In vitro	Peptide-based biosensor fabrication for biomarker Aβ_1–42_	Quantifies the Aβ_1–42_ in artificial CSF and spiked serum samples.	[79]
Au/NiFe2O4@GO-Ch	Hybrid AuNPs	In vitro	Immuno-sensing of Aβ peptide	Aids in quantifying Aβ_1–42_ molecules using DPV.	[80]
Gold urchin and hybrid	Hybrid AuNPs	In vitro	Aβ oligomers detection	Enables the selective and sensitive detection of Aβ oligomers (AβOs), offering promise for AD diagnosis.	[81]
L-glutathione-coated AuNPs	Hybrid AuNPs	In vivo *	Aβ fibrillation	GSH possesses unbound carboxyl and amino groups capable of interacting with Aβ through non-specific electrostatic and hydrogen bonding interactions.	[82]
Gold nanorods (Au NRs) with CeO_2_ NPs	Hybrid AuNPs	In vitro and mice model; 25 mg/kg; Intravenous.	Photothermal therapy of AD	Enhanced intercellular anti-ROS activity.	[83]
HAuNS and AuNR	Functionalized AuNPs	In vitro	Inhibition of Aβ aggregation	Exposing Aβ to irradiation and heating had no impact on its aggregation. Effectively inhibits the Aβ aggregation.	[84]
AuNPs	Functionalized AuNPs	In vitro and Ex vivo model (rat brain homogenate)	Anticholinesterase inhibition	The charge distribution on AChE affects how effectively nanoparticles can inhibit its activity. Even when small NPs aggregate into larger particles, gaps between them still allow AChE molecules to interact and bind.	[85]
Mimo-AuNPs	Functionalized AuNPs	In vitro	Suppression of Aβ aggregation	Mimo-AuNPs induce disassembly by disrupting the β-sheet structure or interacting with the steric zippers within Aβ_1–42_ fibers.	[86]
PFGNP	Functionalized AuNPs	In vitro	Inhibition of Aβ_1–42_ fibrillogenesis	Inhibits the Aβ_1–42_ fibril formation.	[87]
AuNPs	Functionalized AuNPs	In vitro and Ex vivo model (Plasma samples from humans)	Detection of the ADAM10 biomarker	Aids in detecting ADAM10 in diluted plasma, demonstrating a low detection limit and a dynamic linear range.	[88]
Iron oxide-AuNPs	Dual component-Functionalized AuNPs	In vitro	Neuronal Ca^2+^ flux regulation	Enhances intracellular calcium influx and facilitates the cellular uptake of superparamagnetic iron oxide-gold NPs. Induces the upregulation of neural differentiation markers and cell adhesive molecules.	[89]
Gold nanorods	Functionalized AuNPs	In vitro	Disaggregation of the amyloid	GNR-PEG-Ang2/D_1_ was able to cross the BBB with the help of the Ang2 peptide. TEER assays and confocal imaging showed increased tight junction expression, indicating stronger barrier integrity.	[90]
Cysteine-Aβ Peptide-conjugated AuNPs	Functionalized AuNPs	In vitro and Ex vivo model (Plasma samples from humans)	Aβ Aggregation	Cysteine-functionalized Aβ peptides interact with AuNPs, changing peptide conformation and slowing aggregation.	[91]
AuNPs	Functionalized AuNPs	In vitro	Anti-AD	Nanogold aqueous extracts show strong antioxidant and anti-acetylcholinesterase activity. They may cross the BBB, reduce oxidative stress, and improve acetylcholinesterase levels.	[92]

NPs: Nanoparticles; AuNPs: Gold nanoparticles; AD: Alzheimer’s disease; Aβ: Amyloid β; AChE: Acetylcholinesterase; Au@Pt/Au core@shell NPs: Gold Platinum Nanoparticles; Qu@P-80@AuPd: Quercetin-modified gold-palladium nanoparticles; CSF: Cerebrospinal fluid; Au/NiFe_2_O_4_@GO-Ch: Gold nanoparticle/nickel ferrite decorated graphene oxide-chitosan nanocomposite; DPV: Differential pulse voltammetry; GSH: L-glutathione; CeO_2_ NPs: Ceria nanoparticles; ROS: Reactive oxygen species; HAuNS: Hollow gold nanospheres; AuNR: gold nanorods; PFGNP: Polypeptide functionalized gold nanoparticles; ADAM10; A Disintegrin and Metalloproteinase 10; GNR-PEG-Ang2-D_1_: gold nanorods coated with polyethylene glycol and biofunctionalized with peptide and Angiopep-2 peptide; TEER: Trans-endothelial electrical resistance; Cys-Aβ@AuNP: Cysteine-Aβ peptide-conjugated gold nanoparticles; BBB: Blood–brain barrier; SH-SY5Y: Human Neuroblastoma Cell Line; Mimo-AuNPs: Mimosine functionalized gold nanoparticles; * Full text paper is not available, so the information has not been included.

**Table 2:** Recent clinical trials with gold and other nanoparticles for treating neurodegenerative diseases (Based on the data from ClinicalTrials.gov dated 20 August 2025).

Status	Study Title	Classification	Conditions	Interventions	Phase	Details	Identifier
Completed	31P-MRS imaging to assess the effects of CNM-Au8 on impaired neuronal redox state in Parkinson’s disease	Single metal NPs	PD	Drug: Gold nanocrystals	Phase 2	Duration: From 19 December 2019 to 7 June 2021; No of subjects: 30.	NCT03815916 (https://www.clinicaltrials.gov/study/NCT03815916) (accessed 20 August 2025) [104]
Completed	A multi-center, open-label, long-term extension study of CNM-Au8 in patients with stable relapsing multiple sclerosis	Single metal NPs	MS	Drug: CNM-Au8	Phase 2 Phase 3	Duration: From 22 October 2020 to 6 September 2023; No of subjects: 55.	NCT04626921 (https://www.clinicaltrials.gov/study/NCT04626921) (accessed 20 August 2025) [105]
Completed	Therapeutic nano-catalysis to slow disease progression of ALS	Single metal NPs	ALS	Drug: CNM-Au8	Phase 2	Duration: From 19 December 2019 to 13 July 2021; No of subjects: 45.	NCT04098406 (https://www.clinicaltrials.gov/study/NCT04098406) (accessed 20 August 2025) [106]
Active and not recruiting	31P-MRS imaging to assess the effects of CNM-Au8 on impaired neuronal redox state in multiple sclerosis	Single metal NPs	MS	Drug: Gold nanocrystals	Phase 2	Not applicable	NCT03993171 (https://www.clinicaltrials.gov/study/NCT03993171) (accessed 20 August 2025) [107]
Active and not recruiting	An open-label extension for the phase 2 study in early symptomatic amyotrophic lateral sclerosis patients on stable background therapy to assess bioenergetic catalysis with CNM-Au8 to slow disease progression in amyotrophic lateral sclerosis (ALS)	Single metal NPs	ALS	Drug: CNMAu8 (Clene nanomedicine-gold nanocrystal-based compound)	Phase 2	Not applicable	NCT05299658 (https://www.clinicaltrials.gov/study/NCT05299658) (accessed 20 August 2025) [108]
Unknown status	Study of APH-1105 in patients with mild to moderate AD	Functionalized lipid NPs	Mild to moderate AD	Drug: APH-1105	Phase 2	Not applicable	NCT03806478 (https://www.clinicaltrials.gov/study/NCT03806478) (accessed 20 August 2025) [109]
Withdrawn	31P-MRS imaging to assess the effects of CNM-Au8 on impaired neuronal redox state in ALS	Single metal NPs	ALS	Drug: Gold nanocrystals	Phase 2	Not applicable	NCT03843710 (https://www.clinicaltrials.gov/study/NCT03843710) (accessed 20 August 2025) [110]
Terminated	Nanocrystalline gold to treat remyelination failure in chronic optic neuropathy in multiple sclerosis	Single metal NPs	MS	Drug: CNM-Au8	Phase 2	Not applicable	NCT03536559 (https://www.clinicaltrials.gov/study/NCT03536559) (accessed 20 August 2025) [111]

AD: Alzheimer’s disease; PD: Parkinson’s disease; MS: Multiple sclerosis; CNM: Clene Nanomedicine; APH: Aphios.

## Data Availability

No new data were created.

## Abbreviations

↑	Increase
↓	Decrease
4HNE	4-hydroxynonenal
Ab	Antibody
AChE	Acetylcholinesterase
AD	Alzheimer’s disease
ADAM10	A Disintegrin and Metalloproteinase 10
AICD	APP intracellular domain
ALS	Amyotrophic lateral sclerosis
APH	Aphios
ApoE4	Apolipoprotein E4
APP	Amyloid precursor protein
APTMS	Amine (3-aminopropyl) triethoxysilane
Au/NiFe2O4@GO-Ch	Gold nanoparticle/nickel ferrite decorated graphene oxide-chitosan nanocomposite
Au@Pt/Au core@shell NPs	Gold platinum nanoparticles
Au-CeO_2_	Gold-cerium oxide
AuNCs	AuNPs and nanoclusters
AuNPs	Gold nanoparticles
AuNR	Gold nanorods
Aβ	Amyloid beta
AβO	Aβ oligomers
BBB	Blood–brain barrier
BBB-oC	BBB-on-a-Chip
BDNF	Brain-derived neurotrophic factor
BDNF-pCREB-pAkt	Brain-derived neurotrophic factor-phosphorylated cyclic AMP response element-binding protein-phosphorylated protein kinase B
BSA	Bovine serum albumin
C99	Membrane-bound 99 amino acid comprising CTF-β
CA1	Cornu ammonis 1
Ce (AC)_3_	Cerium acetate
CeO_2_ NPs	Ceria (cerium oxide) nanoparticles
CNM	Clene nanomedicine
CNS	Central nervous system
CSF	Cerebrospinal fluid
CTAB	Cetyltrimethylammonium bromide
CTF	Carboxy-terminal end
Cys-Aβ@AuNP	Cysteine-Aβ peptide-conjugated gold nanoparticles
D3.3	d-glutathione-stabilized gold nanoparticles
D-Au	4,6-diamino-2-pyrimidinethiol-coated Au
DG	Dentate gyrus
DMF	Dynamic magnetic field
DPV	Differential pulse voltammetry;
fM	Femtomolar
GBA	Gut–brain axis
GFAP	Glial fibrillary acidic protein
GNR-PEG-Ang2/D_1_	Gold nanorods-polyethylene glycol-angiopep-2 peptide/D_1_ peptide
GNS	Gold nanostar
GO	Graphene oxide
GSH	L-glutathione
GSK-3β	Glycogen synthase kinase-3 Beta
HAuNS	Hollow gold nanospheres
HE-AuNPs	*Heliotropium eichwaldi*-functionalized AuNPs
ih	Iliohypogastric nerve injections
IL-1β	Interleukin-1 beta
IP_3_	Inositol 1,4,5-trisphosphate
K_2_PtCl_4_	Potassium tetrachloropropionate
KLVFF	Pentapeptide fragments (Aβ_16–20_)
LDL	Low-density lipoprotein
MF	Magnetic field
Mimo-AuNPs	Mimosine functionalized gold nanoparticles
miRNA-137	microRNA-137
MS	Multiple sclerosis
NF-κB	Nuclear factor kappa-light-chain-enhancer of activated B cells
NFT	Neurofibrillary Tangles.
NGF-SPIO-Au NPs	Nerve growth factor functionalized superparamagnetic iron oxide-gold NPs
NGFβ	Nerve growth factor-β
NIR	Near-Infrared region
NPs	Nanoparticles
OA	Okadaic acid
PC12	P(heo)C(hromocytoma)12 cells
PD	Parkinson’s disease
PEG	Polyethylene glycol
PEG-COOH	Poly(ethylene glycol) with a Carboxylic Acid group
PFGNP	Polypeptid-functionalized gold nanoparticles
PSEN1	Presenilin 1
PSEN2	Presenilin 2
PTT	Photothermal Therapy
Qu@P-80@AuPd	Quercetin-modified gold-palladium nanoparticles
ROS	Reactive oxygen species
sAPPα	Soluble APPα
SCFAs	Short-chain fatty acids
SHG-44	Shanghai Institute of Biochemistry and Cell Biology-44 (Human malignant glioma cell line)
SH-SY5Y	Human Neuroblastoma Cell Line
SMF	Static magnetic field
STIM1	Stromal interaction molecule-1
STIM2	Stromal interaction molecule-2
TEER	Trans-endothelial electrical resistance
TEM	Transmission electron microscopy
TNFα	Tumor necrosis factor-alpha
ΨMMP	Mitochondrial membrane potential
